# The hero of American and Polish nations: a molecular look at Thaddeus Kosciuszko’s cause of death suggests a contribution of endocarditis caused by Cutibacterium acnes infection

**DOI:** 10.1186/s12934-022-01970-7

**Published:** 2022-11-23

**Authors:** Michał Witt, Miron Tokarski, Ewa Ziętkiewicz, Arleta Lebioda, Maria Szczypek, Wojciech Falkowski, Przemysław Mrozowski, Teresa Kulak, Małgorzata Sobieszczańska, Magdalena Mrugalska-Banaszak, Tomasz Jurek, Tadeusz Dobosz

**Affiliations:** 1 Institute of Human Genetics, Polish Academy of Sciences, Strzeszyńska 32, 60-479 Poznań, Poland; 2 Institute of Forensic Medicine, Wroclaw Medical University, Wrocław, Poland; 3 Museum King’s Castle (Zamek Królewski), Warsaw, Poland; 4 Department of History, University of Wroclaw, Wrocław, Poland; 5 Department and Clinic of Geriatrics, Wroclaw Medical University, Wrocław, Poland; 6 City Museum of Poznań, Poznań, Poland

**Keywords:** *Cutibacterium acnes*, Endocarditis, Heart, Kosciuszko, *Propionibacterium acnes*

## Abstract

The remains of the heart tissue of Thaddeus Kosciuszko have been investigated as the possible cause of disease and death of the hero of Polish and American nations. Three specimens, DNA isolated from scrappings of wax surface, from the surface of a wooden plate, and from the linen cloth that have had contact with the object were subjected to nanosequencing. From the first two, among all reads identified, only one classified as *Propionibacterium acnes* (synonymous current name *Cutibacterium acnes*), had a purported clinical significance. The observed identity between the *P. acnes* sequences and reference was 89–90% consistent with the hypothesis that the identified reads represent the ancient *P. acnes* DNA (aDNA), which underwent fragmentation and sequence changes caused by its long-time presence in the environmental conditions conducive to degradation. We present a reasonable and entirely new hypothesis that the analyzed samples could reflect the presence of the bacteria in the original Kosciuszko’s heart tissue and that the process of *C. acnes* infection was progressing inside the organ (endocarditis), not on its surface (pericarditis) leading to rapid deterioration of health and eventually death. We again point out that normal skin and mucosal membranes commensal, a causative agent of common skin acne, may be associated with various severe organ infections posing a threat to health and life.

## Introduction

Thaddeus Kosciuszko, a Polish general and engineer, the hero of Polish and American nations, was born in 1746 in eastern Poland (at that time called Polish–Lithuanian Commonwealth) and died in 1817 in Soleure (Solothurn), Switzerland. Kosciuszko received an in-depth military education in Warsaw and later in France. Soon after his arrival in North America in 1776 he befriended Benjamin Franklin and Thomas Jefferson and took part in the American Revolutionary War. He was extremely successful as a commander and guided several important military achievements like the fortification of Fort Mercer, West Point, and Philadelphia. Kosciuszko significantly contributed to the American victory at Saratoga, when the defenses along the Hudson River led the Continental Army to final success. Without Kosciuszko’s contributions to the Battle of Saratoga, the Americans might have lost, and France might have never entered the war on the American side. In recognition of his accomplishments, in 1776 he was awarded the rank of colonel by the American Congress being considered equal in terms of knowledge and experience to the Marquis de Lafayette, although undeservedly less renowned [1, 2]. Posthumously, he was commemorated in America with Thaddeus Kosciuszko National Memorial in Philadelphia and with statues in Washington, Boston, Detroit, and many other cities.

Kosciuszko returned from America to Poland in 1784, hoping that the American Revolution could serve as a model for his own country to resist foreign domination and achieve democratic reforms, In 1791, he witnessed the passing of a new Polish constitution, generally being considered as the second modern and complete national constitution in history, the first being that of the United State. In 1794 Kosciuszko was proclaimed commander in chief of Poland and chosen to lead the uprising against Russian invaders (Kosciuszko Insurrection). Despite some military successes, like a victory in the Raclawice battle, this fiery rebellion was ultimately lost; Kosciuszko was wounded and taken prisoner. After being released in 1796 he went again to America. Kosciuszko spent the last years of his life, from 1798, in France and Switzerland [3, 4]. In 1817, after unfortunate falling from a horse, reportedly straight into an icy stream in the vicinity of Vevey on the north shore of Lake Geneva, Kosciuszko contracted a severe cold, developed pneumonia, and supposedly suffered from a fatal stroke caused by his immobility [5, 6]. He died at age 71.

Kosciuszko’s embalmed body was repatriated to Poland in 1819 and put to rest in a sarcophagus among kings and presidents in a national pantheon in Wawel Castle, Cracow. In the tradition of the romantic era, the heart of Kosciuszko after his death was removed from the body, wrapped up in a black silk fabric, put on a wooden plate, and placed in a glass filled with ethanol. It was kept at the Polish National Museum in Rapperswil, Switzerland, tightly closed till 1895 when the conservation fluid was changed into 3% phenol in glycerol. The heart was brought to Warsaw in 1927. After World War II, the majolica vase with the heart, locked in a metal cabinet, was incidentally recovered in 1947 in the ruins of St. John’s Cathedral. The jar was transferred to the King’s Castle (Zamek Królewski) in Warsaw and stored till 1961 when tissue debris have been preserved by dunking in bee wax.

In 2017 the heart, which by this time lost its original shape and integrity, was recovered from wax and re-conserved with 35% ethanol/10% glycerol. During this operation, performed at a current location of the object in the King’s Castle, a portion of wax and of a wooden plate, which had been in close contact with the heart tissue, were collected [7] to be used for molecular identification of the genetic material recovered from the specimens.

## Methods

After scrapping about 100 mg of tissue from the surface of the wax with a spatula, the material was deparaffinized by immersing it two times in 1 ml of xylene (Sigma), for 1 h at room temperature. Next, the residue of xylene was removed by short flushing in absolute ethanol (Chempur). Tissue was digested overnight at 37 °C, in 1 ml of digesting buffer (10 mM Tris, 10 mM EDTANa_2_, 100 mM NaCl, 40 mM DTT, 20 mg SDS, 40 μg Proteinase K; pH = 8.0).

DNA was purified using QIAquick PCR Purification Kit (QIAGEN). After quantification with Quantifiler™ Human DNA Quantification Kit (ThermoFisher Scientific), the DNA isolate was prepared for sequencing using MinION Next Generation Sequencing Platform (Oxford Nanopore Technologies, Oxford UK, ONT) according to the manufacturer’s procedure [8–10].

DNA library preparation was performed using a 1D PCR barcoding-by-ligation kit (SQK-LSK108) (ONT). The whole volume of the extracted DNA—45 µl—was used for dA-tailing and end-repair with Ultra II End-prep enzyme mix (New England Biolabs—NEB). After incubation, the sample was cleaned-up with AMPure XP beads (Beckman Coulter). Next, 30 µl of the eluate was used for Adapter ligation with 50 µl Blunt/TA Ligation Master Mix (NEB) and 20 µl of Adapter Mix (ONT). After incubation at RT, 100 µl of the reaction was used for bead-binding with AMPure XP beads and 140 µl of Adapter Bead Binding Buffer (ONT). Finally, the sample was eluted with 15 µl of Elution Buffer (ONT). To run the sequencing process, we utilized the R9.4 SpotOn flow cell (ONT), which was primed according to the manufacturer’s instructions. In the last step, 12 µl of DNA library was mixed with 25.5 µl of Library Loading Beads, 35 µl of Running Buffer, and 2.5 µl of nuclease-free water and transferred to the sequencing cell. The 48-h sequencing run was executed using the MinKNOW software for primary data acquisition (ONT).

The second sample, shavings from the surface of the wooden plate supporting the heart, was handled by a different operator. DNA extraction, library preparation, and sequencing (in a new flow cell) were conducted in the same way as from the wax sample.

The third sample was isolated from a specimen kept in the City Museum in Poznań. It was a fragment of the linen cloth, with which Kosciuszko’s heart was wiped up in 1897 after being removed from ethanol, before transfer to glycerol/phenol conservation fluid. Traces of biological material were extracted from the cloth using a non-destructive method of isolation of DNA from museum objects; DNA was purified and sequenced using the same method as described above.

## Results

EPI2ME platform (ONT) was used to classify the nanopore sequencing reads in the material from both samples. In the wax sample, Fastq WIMP Rev 2.1.0 workflow was applied; during the process, base-calling with QScore filter [rev. 3.8.0] analyzed 253 reads with the Average Quality Score of 9.38, and an average sequence length of 405 bp (total yield 102.5 KBp). In the second sample, of wood shavings, Fastq WIMP Rev 3.2.2 workflow was used; base-calling with QScore filter [rev. 3.7.2] analyzed 30 reads with the Average Quality Score of 8.35; the average sequence length was 350 bp (total yield 10.5 KBp).

In the DNA sample isolated from bee wax, sixteen known sequences were identified (and 237 remained unclassified). Eleven reads were from *H. sapiens*, one from a dog, one from an insect, and three from bacteria (*Escherichia coli*, *Janthinobacterium* sp., and *Propionibacterium acnes*. Seven known sequences (and 23 unclassified) were read from the wooden plate sample; four reads were classified as of human origin, two were from *E. coli,* and one from *P. acnes*. Nanosequencing of DNA purified from the linen cloth, with which Kosciuszko’s heart had been wiped up, revealed several hundred reads, all of human origin. Importantly, no signs of fibrin flakes typical of “frosted heart”, a hallmark of fibrinous pericarditis, were found on the cloth [11, 12]. These sequences originated possibly either from Kosciuszko’s genome or, most probably, from random human contamination.

Only one of the sequences from each of the wax and wood samples, classified as *Propionibacterium acnes* (synonymous current name *Cutibacterium acnes*), had a purported clinical significance (see Discussion). Of note, sequences of *Salmonella enterica* serotype Typhi, the cause of typhoid fever alleged by some sources as responsible for a fatal disease of Kosciuszko [5],were not identified in the material studied.

**Fig. 1:**
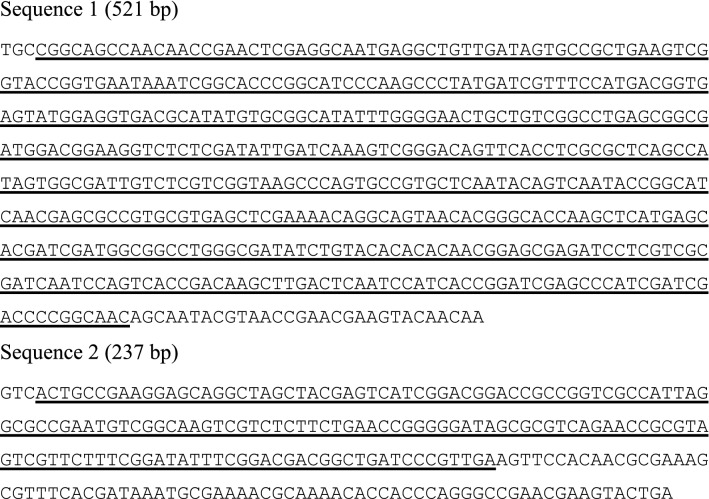
*P. acnes* sequences from wax (1) and wood (2); fragments classified as corresponding to *P. acnes* are underlined (see Fig. 2)

*P. acnes* sequences obtained from the wax and wooden plate samples (see Fig. 1A, B) mapped to different regions of the bacterial genome, corresponding to fragments of the amidohydrolase and ribosome-associated GTPase genes, respectively. They both were classified as derived from the same strain of bacteria, *P. acnes* TypeIAP2.acn31.

**Fig. 2:**
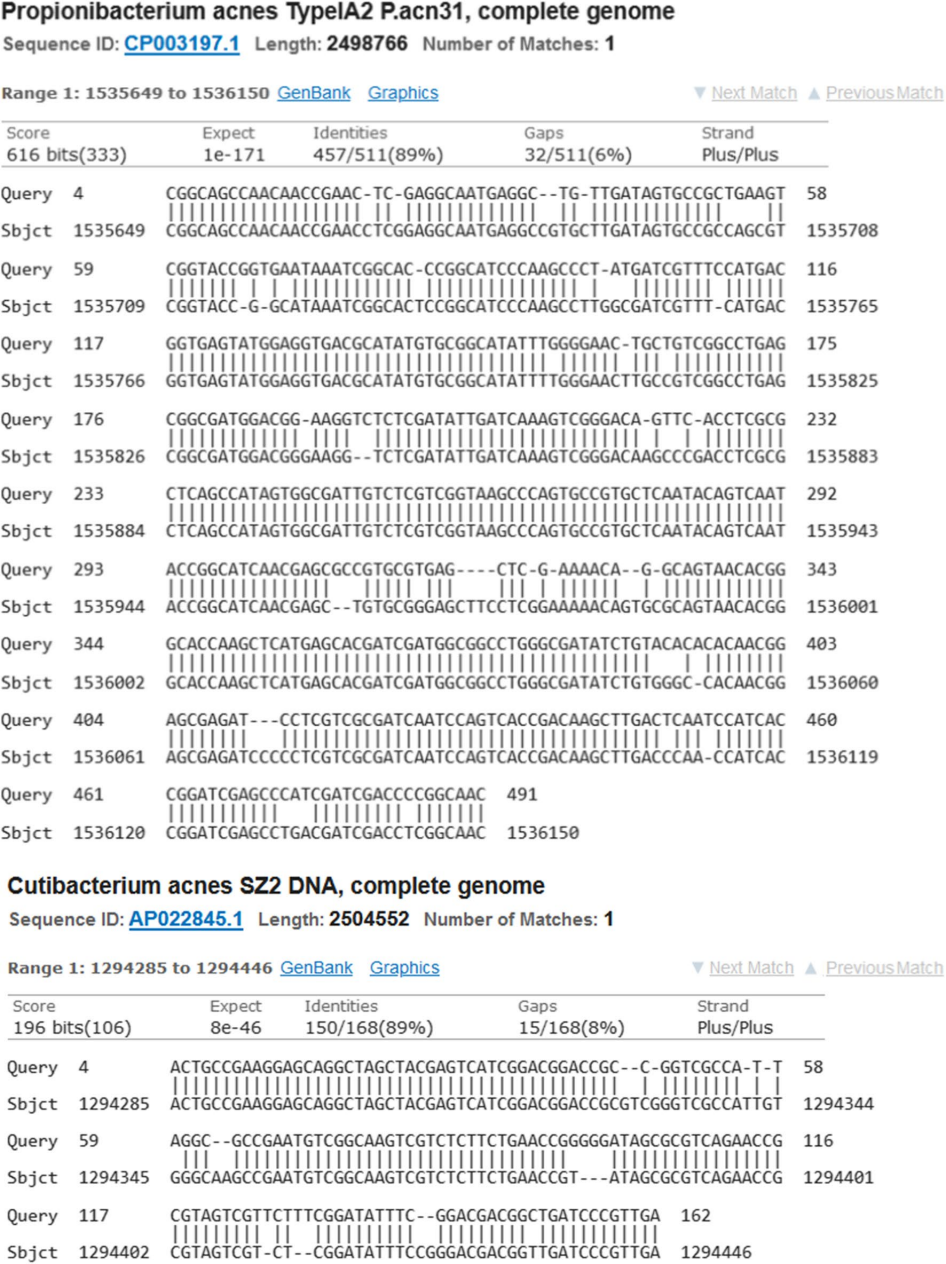
BLASTN alignments of the *P. acnes* sequences

The wax sequence was similar to the reference along 487 bp (94% of the whole read), the wood sample only along 150 bp (67%). The identity of the aligned fragments was over 89% in both samples, with several randomly distributed mismatches and indels (see Table 1 and Fig. 2).

**Table 1:** *P. acnes* TypeIAP2.acn31

Source of query sequence	Organism	Read length	Query cover	Alignment length	Identity (BLASTN)	Mapping
Wax sample	*P. acnes* TypeIAP2.acn31	521 bp	487 bp (94%)	511 bp	459/511 (89.4%)	Amidohydrolase gene
Wood shavings	*P. acnes* TypeIAP2.acn31	237 bp	159 bp (67%)	168 bp	151/168 (89.9%)	Ribosome-associated GTPase gene

Sequence 1 (521 bp).

TGCCGGCAGCCAACAACCGAACTCGAGGCAATGAGGCTGTTGATAGTGCCGCTGAAGTCG

GTACCGGTGAATAAATCGGCACCCGGCATCCCAAGCCCTATGATCGTTTCCATGACGGTG

AGTATGGAGGTGACGCATATGTGCGGCATATTTGGGGAACTGCTGTCGGCCTGAGCGGCG

ATGGACGGAAGGTCTCTCGATATTGATCAAAGTCGGGACAGTTCACCTCGCGCTCAGCCA

TAGTGGCGATTGTCTCGTCGGTAAGCCCAGTGCCGTGCTCAATACAGTCAATACCGGCAT

CAACGAGCGCCGTGCGTGAGCTCGAAAACAGGCAGTAACACGGGCACCAAGCTCATGAGC

ACGATCGATGGCGGCCTGGGCGATATCTGTACACACACAACGGAGCGAGATCCTCGTCGC

GATCAATCCAGTCACCGACAAGCTTGACTCAATCCATCACCGGATCGAGCCCATCGATCG

ACCCCGGCAACAGCAATACGTAACCGAACGAAGTACAACAA

Sequence 2 (237 bp).

GTCACTGCCGAAGGAGCAGGCTAGCTACGAGTCATCGGACGGACCGCCGGTCGCCATTAG

GCGCCGAATGTCGGCAAGTCGTCTCTTCTGAACCGGGGGATAGCGCGTCAGAACCGCGTA

GTCGTTCTTTCGGATATTTCGGACGACGGCTGATCCCGTTGAAGTTCCACAACGCGAAAG

CGTTTCACGATAAATGCGAAAACGCAAAACACCACCCAGGGCCGAACGAAGTACTGA

## Discussion

The observed identity between the *P. acnes* sequences of DNA extracted from wax and wooden plate and the corresponding fragments of *P. acnes* TypeIAP2.acn31 reference was 89% and 90%, respectively. Erroneous sequence readings might play a role in lowering the identity; the accuracy of reads claimed by the vendor was 95–97%. If corrected for the technical errors, the actual identity values for both DNA fragments would be 94% and 95%, respectively.

Theoretically, the differences with respect to the reference sequence could point to the isolated sequences originating from a *P. acnes* strain not deposited in the database. However, repeated blasting against the GenBank resources did not reveal any entries, which would represent even remote similarity to bacterial species other than *P. acnes* TypeIAP2.acn31. We hypothesize that the identified reads represent the ancient *P. acnes* DNA (aDNA), which underwent fragmentation and sequence changes caused by its long-time presence in the environmental conditions conducive to degradation. In this context, it should be emphasized that some of the human and *E.coli* sequences reads from the wood sample displayed higher compliance with their reference sequences (over 92%) and were much longer (over 1.2 kb), which may suggest that, in contrast to *P. acnes*, they represented recent contamination.

In our opinion, the *P. acnes* sequences identified in the DNA isolated from the analysed samples could reflect the presence of the bacteria in the original heart tissue. We exclude the possibility of ancient post-mortem contamination for the following reasons. The *P. acnes* sequences were recovered both from the wax and from the wood containing the debris of the heart tissue, but not from the cloth used to wipe the external surface of the heart, which at that time was not decomposed. The amount of the DNA was sufficient to read the sequence without relying on nested PCR, which has to be applied in case of extremely low titers of contaminating bacterial material. According to the autopsy record, Kosciuszko’s heart, after being extracted from the corpse, was immediately placed in ethanol, which prevented the growth of potential contaminating bacteria. After 78 years in ethanol, the heart was transferred directly to a new conservation fluid containing 3% phenol, a highly potent bactericide; wax pieces with heart debris even today emitted a foul odor of phenol.

*C. acnes* is a Gram-positive, slow-growing coccobacillus that is normal skin and mucosal membranes commensal. Commonly known as a causative agent of skin acne, it has been associated with various severe infections, both native and postoperative, like endocarditis, pericarditis, osteomyelitis, arthritis, spondylodiscitis, endophthalmitis, and brain abscess [13]. The ability of *C. acnes* to cause deep-seated infections, despite its presumed low virulence, is attributed to its capability to form biofilms [14]. Critical heart complications of *C. acnes* infection include constrictive pericarditis, tamponade, and left ventricular pseudoaneurysm, or acute endocarditis with free-floating thrombi [15]. Endocardial complications involve almost exclusively males (most likely due to a greater number of sebaceous glands), of age 65+. The mortality is relatively high (15% to 27%) [13]. Risk factors include: pneumococcal pneumonia as a triggering event, purulent dermatitis e.g. due to injuries (specifically under the arm and around the shoulder), and lowered overall immunity [16]. The year of Kosciuszko's death, 1817, was conducive to immune imbalance due to severe D3 avitaminosis, as it was the famous second consecutive European "year without summer", after the eruption of the Mount Tambora volcano in Indonesia [17].

The presented data, support a hypothesis that the process of *C. acnes* infection in Kosciuszko’s heart was progressing inside the organ (endocarditis), not on its surface (pericarditis). This is consistent with the canonical version of pneumonia being the alleged cause of Kosciuszko’s death. In the nineteenth century, differential diagnosis of severe pneumonia, acute endocarditis, and thromboembolia was not available. In fact, one does not preclude the other, since the cerebral stroke and/or pulmonary thromboembolia can readily follow *C. acnes* endocarditis. Incidence of bacterial endocarditis in the pre-antibiotic era was as high as 1:250, in our time being lowered to 1:18,000. Clinical presentation of the disease involves fever, chills, shortness of breath, malaise, tachycardia, arrhythmia, pleuritic chest pain, dyspnoea, and lower limb swelling. Such nonspecific but acute symptoms, which may resemble bacterial pneumonia, occurred in Kosciuszko in the period preceding his death [18, 19].

Our novel finding leads us to claim that it is highly likely that specifically acute endocarditis resulting from the earlier infection with commensal bacteria *C. acnes* could have contributed to the fatal outcome of the ailment and, eventually, the death of Thaddeus Kosciuszko. This confirms reports that normal skin and mucosal membranes commensal may be associated with various severe organ infections constituting a significant health problem.

## Data Availability

Not applicable.
